# Elaboration of New Materials Using Hydrothermal Methods

**DOI:** 10.3390/ma15217792

**Published:** 2022-11-04

**Authors:** Daniel Ursu, Cristian Casut, Marinela Miclau

**Affiliations:** 1 National Institute for Research and Development in Electrochemistry and Condensed Matter, Dr. A. Păunescu-Podeanu Street No. 144, 300569 Timişoara, Romania; 2 Faculty of Physics, West University of Timisoara, Bd. V. Pârvan nr. 4, 300223 Timisoara, Romania

“Elaboration of New Materials Using Hydrothermal Methods” is a new and open Special Issue of *Materials*, which aims to publish original research and review papers on that present state-of-the-art advances in the research on the hydrothermal synthesis of new materials. This Special Issue also hopes to inspire a different perspective that will in turn make hydrothermal techniques—such as the continuous production of materials, hydrothermal recycling technology, and the modeling and simulation of hydrothermal synthesis—more economic.

The hydrothermal method is still a “black box” technology based on the crystallization of the materials directly from an aqueous solution by the control of thermodynamic (temperature, pressure, pH of the solution and chemical composition of the precursors) and non-thermodynamic variables. Based on the unique pressure–temperature interaction of a hydrothermal solution, the control of the rate and uniformity of nucleation and growth allows the size, morphology, stoichiometry, polymorphism, metastable phases, and aggregation control of obtained materials to be accurately designed. Additionally, the ability to make new materials is conditioned by understanding the solution thermodynamics of the aqueous medium and the prediction of the phase equilibrium and mechanisms of crystallization via thermodynamic modeling of the hydrothermal systems.

Hydrothermal research was popularized by geologists in the mid-nineteenth century and focused on laboratory simulations of natural hydrothermal phenomena. The continuous development of contemporary advanced science and technology has led to a greater diversification and complexity of hydrothermal technology covering several interdisciplinary branches of science and is not only limited to the crystal growth [1]. Thus, hydrothermal methods can be considered as part of important technologies, such as nanotechnology and advanced materials technology characterized by a highly interdisciplinary subject, as well as a technique used by physicists, chemists, ceramists, materials scientists, and engineers.

The research focus of this Special Issue, “Elaboration of New Materials Using Hydrothermal Methods”, includes but is not limited to the following topics: hydrothermal synthesis, metastable phase, supercritical hydrothermal growth, continuous-flow hydrothermal synthesis, modeling and simulation of hydrothermal synthesis, hydrothermal carbonization, and hydrothermal recycling technology.
